# High-Throughput RNA Sequencing Analysis of Plasma Samples Reveals Circulating microRNA Signatures with Biomarker Potential in Dengue Disease Progression

**DOI:** 10.1128/mSystems.00724-20

**Published:** 2020-09-15

**Authors:** Jaya Saini, Bhaswati Bandyopadhyay, Abhay Deep Pandey, V. G. Ramachandran, Shukla Das, Vikas Sood, Arup Banerjee, Sudhanshu Vrati

**Affiliations:** a Regional Center for Biotechnology (RCB), Faridabad, India; b Calcutta School of Tropical Medicine (STM), Kolkata, India; c Translational Health Science and Technology Institute (THSTI), Faridabad, India; d University College of Medical Sciences (UCMS) & Guru Teg Bahadur (GTB) Hospital, Delhi, India; e Department of Biochemistry, Jamia Hamdard, New Delhi, India; Harvard Medical School

**Keywords:** dengue, RNA sequencing, circulating miRNA, plasma microRNA

## Abstract

The circulating microRNA (miRNA) profile has been widely used for identifying potential biomarkers against viral infections. However, data on circulating microRNA expression patterns in dengue patients are scanty. Considering the impact of severity caused by dengue infection, circulating miRNA profiles in plasma of dengue patients may prove to be valuable for developing early prognostic markers for the disease severity. Here, we described an in-depth analytical study of small RNA sequencing data obtained from the plasma of 39 dengue patients. Integrating bioinformatics and *in vitro* studies, we identified differentially expressed miRNAs (DEMs) (log_2_ fold change ≥1.5, *P* < 0.05) associated with dengue disease progression. In comparing miRNA expression pattern with the follow-up samples, nine miRNAs were found to exhibit an altered expression that could distinguish between severe dengue and the convalescent patients. To understand the abundance and specificity of the DEMs in the context of dengue infection and disease progression, eight top-hit DEMs were further validated in the dengue virus-infected cell lines as well as in the patient’s plasma and peripheral blood mononuclear cells (PBMCs) using the quantitative reverse transcription-PCR (qRT‐PCR) method. Importantly, receiver operating curve analysis further confirmed that the plasma expression pattern of hsa-miR-122-5p could differentiate between different stages of dengue infection (area under the concentration-time curve [AUC] = 0.792), and dengue-negative patients with other febrile illnesses (AUC = 0.984). The *in silico* analysis of DEM target genes suggested an enrichment of the pathways associated with metabolism and inflammation. Our study gives a global view of miRNA expression in the plasma from dengue patients and provides a precious resource of candidate miRNAs involved in dengue infection and disease progression.

**IMPORTANCE** Dengue virus (DENV) infection usually causes dengue fever (DF) with flu-like illness affecting infants, young children, and adults. The DF occasionally evolves into a potentially lethal complication called dengue severe (DS) leading to a rapid fall in platelet count along with plasma leakage, fluid accumulation, respiratory distress, and severe bleeding. The diverse clinical spectrum of dengue disease, as well as its significant similarity to other febrile viral illnesses, makes early identification more challenging in this high-risk group. microRNAs (miRNAs) are small (∼19 to 21 nucleotides [nt] in length), noncoding RNAs, extremely stable and easily detectable in the plasma; thus, they have potential as biomarkers for diagnosing and monitoring human diseases. This study provides a comprehensive analysis of miRNAs circulating in plasma of dengue virus-infected patients and identifies the miRNA signatures that have biomarker potential for dengue infection and disease progression.

## Abstract (precis)

Dengue virus (DENV) infection usually causes dengue fever (DF) with flu-like illness affecting infants, young children, and adults. The DF occasionally evolves into a potentially lethal complication called dengue severe (DS) leading to a rapid fall in platelet count along with plasma leakage, fluid accumulation, respiratory distress, and severe bleeding. The diverse clinical spectrum of dengue disease, as well as its significant similarity to other febrile viral illnesses, makes early identification more challenging in this high-risk group. microRNAs (miRNAs) are small (∼19 to 21 nucleotides [nt] in length), noncoding RNAs, extremely stable and easily detectable in the plasma; thus, they have potential as biomarkers for diagnosing and monitoring human diseases. This study provides a comprehensive analysis of miRNAs circulating in plasma of dengue virus-infected patients and identifies the miRNA signatures that have biomarker potential for dengue infection and disease progression.

## INTRODUCTION

Dengue virus infection is recognized as one of the most important mosquito-borne human diseases of the 21st century. The global incidences of dengue infection have now increased enormously, and an estimated 50 to 100 million cases of dengue infection are reported annually from more than 100 tropical and subtropical countries of the world (1, 2). For more than a decade, dengue fever (DF) has been one of the leading causes of hospitalization and deaths after diarrheal and respiratory infections in Southeast Asia, mostly affecting children. Clinically, dengue can present as a mild febrile illness or a severe life-threatening disease known as dengue hemorrhagic fever (DHF). The hallmark of DHF is increased vascular permeability and consequent plasma leakage, leading to rash, bleeding, circulatory collapse, and shock. The morbidity and mortality of DHF are primarily driven by vascular leakage and its resulting complications. The estimated number of annual global DF cases is between 20 and 30 million, and DHF cases are about 2,00,000. DF and DHF are widely prevalent in India, and all four serotypes of dengue virus (DENV) are involved with various degrees of disease severity (3). The disease has been reported from 18 states and union territories since 1996, with about 450 million of the population at risk (4). Identification of prognostic markers of severe dengue illness can potentially improve patient triage, allocating suitable treatment, i.e., selecting a rational treatment comprising antivirals and immune-modulating therapies, and overall better patient management.

microRNAs (miRNAs) are highly conserved RNAs that can precisely regulate gene expression by targeting mRNAs. These are small (∼19 to 21 nucleotides [nt] in length) single-stranded, noncoding RNAs, which are synthesized inside the cells, encapsulated within small vesicles called exosomes, and later released in the circulation. Thus, miRNAs in circulation are resistant to extracellular nuclease activity, making them extremely stable in extracellular fluids such as plasma (5). The circulating miRNAs are widely used as biomarkers associated with diverse diseases and have been useful in diagnosing and monitoring human diseases (6–10). Besides, the circulating miRNA profiles also provide an insight into the disease mechanisms (11, 12).

Considering the pivotal role of miRNAs in infection and disease progression, we proposed that certain circulating miRNAs may have a significant role in the outcome of dengue infection, and the levels of some of the miRNAs in the blood may serve as an indicator of disease progression. To our knowledge, the information on microRNA expression patterns in plasma of dengue patients is scanty, and nothing is known on how they could control some of the mRNAs and proteins that are dysregulated during the infection. These pieces of information on a cohort of dengue patients may prove to be valuable for developing early prognostic markers for the disease severity. A study, therefore, was conducted to catalog the miRNAs circulating in the plasma of the patients during dengue infection and identify the miRNAs associated with disease progression. To achieve it, we have used a high-throughput small RNA sequencing approach on plasma samples from dengue patients with varying disease severity and compared the data between the different clinical groups of dengue patients. We also sequenced the miRNA plasma samples from the patients followed up on at least two time points and explored the potential biological function of identified candidate miRNAs using *in silico* analysis.

The study provides a global view of the miRNA expression in the plasma from dengue patients and presents a valuable resource on miRNAs that might be involved in the dengue disease manifestation and progression.

## RESULTS

### Shared and unique circulating miRNA signatures in different grades of dengue patients.

**FIG 1:**
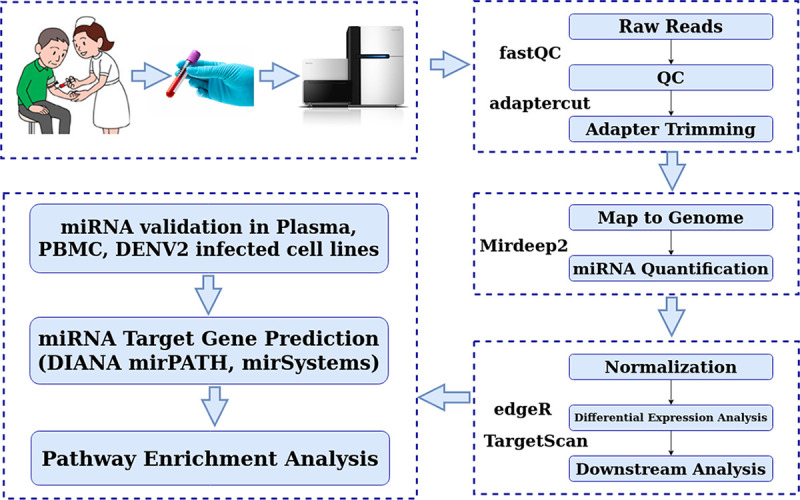
Flow chart depicting the sample details and the analysis pipelines of small RNA sequencing.

**FIG 2:**
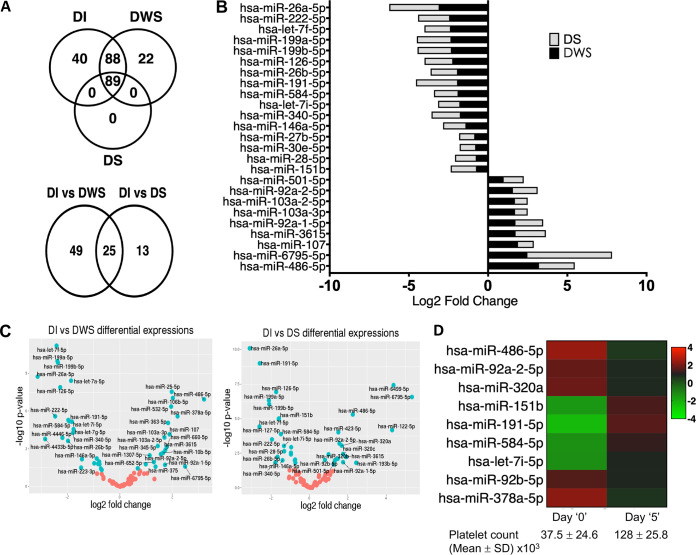
Plasma miRNA profiling of dengue-infected patients. (A) Venn diagram of common and unique miRNAs detected in DI, DWS, and DS patients (upper panel). The number of differentially expressed miRNAs in DWS and DS groups compared to DI (lower panel). (B) Expression pattern of 25 miRNAs differentially expressed in DWS or DS versus DI patients shown as log_2_ fold change. (C) Volcano plots showing changes in the expression levels of miRNAs between DWS and DI groups and between DS and DI groups. The log_2_ fold change is depicted on the *x* axis, and log_10_ *P* values are shown on the *y* axis. The *P* values were calculated using the Student *t* test. (D) Heatmap of 9 miRNAs differentially expressed in plasma of the DS group of patients and the convalescent patients.

Using the high-throughput small RNA sequencing methods followed by the *in silico* analyses, we compared the levels of circulating miRNAs between patients with uncomplicated dengue infection (DI; *n* = 9), dengue with a warning sign (DWS; *n* = 14), and dengue severe (DS; *n* = 16). Detailed steps of data analysis were schematically represented in Fig. 1. Among the 239 miRNAs detected in two-thirds of the samples, 89 miRNAs were present in all three dengue groups (DI, DWS, and DS) (Fig. 2A). We performed differential expression analysis of miRNAs between DI and DWS, and then between DI and DS. We retrieved the expressed reads of these 89 miRNAs and subjected them to analysis using the edgeR Bioconductor package to identify differentially expressed miRNAs (DEMis) (13). The results were further sorted using a *P* value of ≤0.05 and log fold change of >0.5. We found 74 differentially expressed miRNAs in DWS versus DI, and 38 in DS versus DI, comparisons (Table 1). Out of the 89 shared miRNAs, 25 were differentially expressed in the DWS and DS groups compared with the DI group (Fig. 2A, lower panel). The differential expression level of these 25 miRNAs in the DWS and DS patients is shown in Fig. 2B. Further, volcano plots were depicted to identify the miRNAs that had the most significant fold differences as well as high statistical significance between the two groups, DWS and DS, compared with the DI group (Fig. 2C).

**TABLE 1:** Clinical parameters of dengue patients and pattern of different microRNAs detected in their plasma

Patient samples used for discovery				
	DI	DWS		DS
No. of plasma samples	9	14		16
Age in years (mean ± SD)	24 ± 8	18 ± 6		20 ± 5
Platelet count (mean ± SD) × 10^3^	104 ± 41	29 ± 5		15 ± 3
Duration of fever (days)	1–3	3–5		3–6
Serotype (DENV-1/2/3)	2/5/2	5/8/1		2/12/2

Patient samples used for validation				
	OFI	DI		DS
No. of plasma samples	7	14		12
Age in years (mean ± SD)	28 ± 06	23 ± 09		20 ± 07
Duration of fever (days) at the time of recruitment	1–4	2–5		3–6
Platelet count (mean ± SD) × 10^3^	95 ± 30	110 ± 55		20 ± 4
Serotype (DENV-1/2/3)	0/0/0	3/10/1		2/10/0

Follow-up patients				
	Age in years (mean ± SD)	Platelet count (mean ± SD) × 10^3^ Day 0		Platelet count (mean ± SD) × 10^3^ Day 5
Group-1 (*n* = 4)	22 ± 8	116 ± 43.8		57.6 ± 13.9
Group-2 (*n* = 5)	25 ± 10	37.5 ± 24.6		128 ± 25.8

miRNA RNA-seq analysis				
	DI	DWS		DS
No. of miRNAs detected	603	668		318
Range of miRNAs detected	138–331	151–466		68–214
Total no. of detectable miRNAs in at least two-thirds of samples	217	199		89

Differentially expressed miRNAs (DEMis)				
	DI vs DWS		DI vs DS	
No. of common expressed miRNAs	177		89	
No. of DEMis (lfc >0.5, *P* value <0.05)^*a*^	74		38	
Upregulated	40		21	
Downregulated	34		17	

a lfc, log fold change.

### Circulating microRNA signature associated with dengue disease progression.

To understand the circulating miRNA signature pattern associated with dengue disease progression, we checked the 25 shared miRNAs that were differentially expressed in the DWS and DS groups compared with the DI group. We observed that the levels of 11 miRNAs were significantly upregulated in the DWS and DS groups, whereas the levels were downregulated for 14 other miRNAs (Table 2). Moreover, six miRNAs were exclusively significantly upregulated, and seven miRNAs were downregulated in the DS group, thus possibly associated with severe dengue manifestation (see Table S1 in the supplemental material). Interestingly, miR-6499 and miR-122 levels were found to be significantly upregulated in plasma samples obtained from the DS patients. Analysis of data from the follow-up patients (Table S2) indicated that the expression of 9 differentially expressed miRNAs (miR-486-5p, miR-92a-5p, miR-320a, miR-151b, miR-191-5p, miR-584-5p, let7i-5p, 92b-5p, and 378a-5p) strongly correlated with dengue disease progression (Fig. 2D). Four of them (miR-486-5p, miR-92a-5p, miR-320a, and miR-191-5p) were differentially expressed in both the DWS and DS groups compared to the DI group (Table 2).

**TABLE 2:** List of 25 common miRNAs that are differentially expressed in the DWS and DS groups compared to the DI group^*a*^

DEMis	DWS vs DI				DS vs DI			
	Log_2_FC	LogCPM	*P* value	FDR	Log_2_FC	LogCPM	*P* value	FDR
hsa-miR-486-5p_MIMAT0002177	3.439916	20.11742	1.29E−08	5.70E−07	2.252847	19.46	4.76E−06	5.30E−05
hsa-miR-6795-5p_MIMAT0027490	2.17638	3.277031	0.038977	0.097169	5.334587	6.387649	2.18E−07	3.88E−06
hsa-miR-107_MIMAT0000104	2.066268	13.02199	1.21E−05	0.000107	0.993164	12.51048	0.004093	0.015513
hsa-miR-92a-1-5p_MIMAT0004507	1.965585	16.68253	0.00691	0.022239	1.74442	16.91278	0.013092	0.034271
hsa-miR-3615_MIMAT0017994	1.960274	9.482536	0.000145	0.000893	1.90729	9.966612	0.005355	0.019062
hsa-miR-103a-3p_MIMAT0000101	1.86487	13.05823	2.54E−05	0.000196	1.86487	13.05823	2.54E−05	0.000196
hsa-miR-103a-2-5p_MIMAT0009196	1.864372	13.05834	2.55E−05	0.000196	0.801697	12.57553	0.010653	0.029628
hsa-miR-92a-2-5p_MIMAT0004508	1.805098	16.69202	0.000632	0.003022	1.566943	16.95568	0.000601	0.003343
hsa-miR-501-5p_MIMAT0002872	1.136966	8.584412	0.008316	0.026284	1.258537	8.893461	0.020494	0.047999
hsa-miR-320b_MIMAT0005792	1.045768	11.86418	0.046535	0.112733	1.466634	12.5178	0.007519	0.024786
hsa-miR-378a-5p_MIMAT0000731	2.304232	16.26194	0.031883	0.084618	2.59451	17.08396	0.005104	0.000713
hsa-miR-22-5p_MIMAT0004495	0.835418	15.42198	0.032508	0.084618	−0.74889	14.72514	0.010064	0.029628
hsa-miR-28-5p_MIMAT0000085	−0.67133	11.33542	0.03714	0.09391	−1.30978	11.41947	0.002196	0.00977
hsa-miR-146a-5p_MIMAT0000449	−1.26751	8.873602	0.017973	0.051311	−1.40153	9.164429	0.010017	0.029628
hsa-miR-340-5p_MIMAT0004692	−1.59211	6.897967	0.000778	0.003378	−1.78721	7.326584	0.014689	0.036315
hsa-let-7i-5p_MIMAT0000415	−1.60672	11.72993	0.000146	0.000893	−1.33609	12.26091	0.000921	0.004553
hsa-miR-26b-5p_MIMAT0000083	−1.76477	7.602164	0.00195	0.00767	−1.66268	8.015003	0.005593	0.019147
hsa-miR-584-5p_MIMAT0003249	−1.7761	10.32449	3.85E−05	0.000262	−1.48971	10.8137	0.00014	0.00089
hsa-miR-191-5p_MIMAT0000440	−1.82956	11.69786	3.39E−05	0.00024	−2.58795	11.80813	9.51E−10	4.23E−08
hsa-miR-126-5p_MIMAT0000444	−2.079	12.24443	9.32E−07	1.49E−05	−1.74087	12.65655	1.46E−07	3.24E−06
hsa-miR-199b-5p_MIMAT0000263	−2.18587	9.579234	1.08E−08	5.70E−07	−2.09462	9.947405	9.58E−07	1.22E−05
hsa-miR-199a-5p_MIMAT0000231	−2.20719	9.657065	8.62E−09	5.70E−07	−2.12116	10.02004	5.30E−07	7.86E−06
hsa-let-7f-5p_MIMAT0000067	−2.22172	11.20618	5.27E−09	5.70E−07	−1.61253	11.72673	1.10E−05	9.80E−05
hsa-miR-222-5p_MIMAT0004569	−2.30126	8.366319	1.43E−05	0.00012	−1.95958	8.822605	0.000317	0.00188
hsa-miR-26a-5p_MIMAT0000082	−2.95341	11.29419	2.34E−08	6.90E−07	−3.1114	11.5445	7.21E−11	6.42E−09

a Abbreviations: FC, fold change; CPM, counts per minute; FDR, false-discovery rate.

10.1128/mSystems.00724-20.2 TABLE S1 DS group-specific differentially expressed miRNAs. Download Table S1, DOCX file, 0.02 MB. Copyright © 2020 Saini et al. 2020 Saini et al. This content is distributed under the terms of the Creative Commons Attribution 4.0 International license.

10.1128/mSystems.00724-20.3 TABLE S2 List of miRNAs identified in follow-up patients and their expression inversely correlated with expression observed in DWS and DS patients. Download Table S2, DOCX file, 0.02 MB. Copyright © 2020 Saini et al. 2020 Saini et al. This content is distributed under the terms of the Creative Commons Attribution 4.0 International license.

Next, we used the quantitative reverse transcription-PCR (qRT-PCR) method to validate the expression of the dysregulated miRNAs seen above in the different dengue groups. To focus on the miRNAs most likely related to dengue infection, we selected candidate miRNAs from the small high-throughput RNA sequencing analysis (RNA-seq) data based on the following criteria: demonstrating >1.0-log-fold-higher or −1.0-log-fold-lower expression between any two out of the three dengue patient groups and giving priority to choosing miRNAs that may be present both in the DWS and DS groups.

For performing a precise biofluid miRNA analysis, the method adopted for data normalization is critical. There is no consensus for the best reference gene to be used to normalize the qRT-PCR data in biological fluid samples. Initially, external synthetic miRNA molecules called spike-ins were used for normalization, but in recent times, identifying and using a specific endogenous miRNA as a reference are suggested (14–17). The best way to normalize the plasma miRNAs is to use an endogenous host miRNA, which is stable, abundant, and detectable without any significant variation in its expression status across the various sample groups. Several microRNAs, e.g., miR-423, miR-16, miR-106b-5p, etc., have been used in the past as a reference (18). We checked our RNA-seq data and found that miR-423 and miR-16 were detectable in all samples with log fold change of <1.0 and *P* value of >0.05. Further validation of these two miRNAs was, therefore, done across the various sample groups. The qRT-PCR cycle threshold (*C_T_*) values for these two miRNAs in the plasma samples were plotted (Fig. S1). We found that miR-16 expression changed significantly in the DS cases, whereas there were no statistical differences in the miR-423-5p *C_T_* values between the control other febrile illness (OFI) patients and the DI and DS patients. Therefore, we used miR-423 as the reference for normalizing the expression data of different miRNAs.

10.1128/mSystems.00724-20.1 FIG S1 Comparative analysis of cycle threshold (*C_T_*) values for miR-423-5p (A) and miR-16-5p (B) in plasma of OFI, DI, and DS patients. **, *P* < 0.005. ns, nonsignificant. Download FIG S1, PDF file, 0.2 MB. Copyright © 2020 Saini et al. 2020 Saini et al. This content is distributed under the terms of the Creative Commons Attribution 4.0 International license.

**FIG 3:**
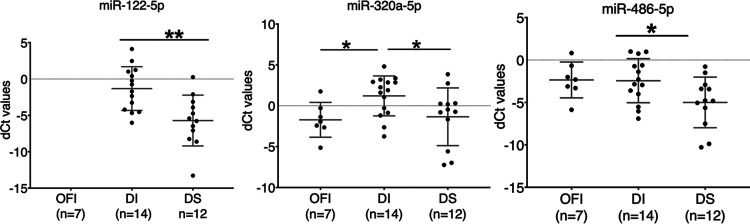
Circulating miRNA levels in different groups of dengue patients. RNA was isolated from the plasma of the patients, and levels of miR-122-5p, miR-320a-5p, and miR-486-5p were quantified by qRT-PCR. miR-423-5p was used as the endogenous reference miRNA to normalize the test miRNAs. The delta-*C_T_* (d*C_T_*) value of each sample was plotted on the *y* axis. The data were analyzed using the unpaired Student *t* test. Mean values are indicated with a line. *, *P* < 0.05; **, *P* < 0.01.

Based on their expression pattern in the DWS and DS patients in the RNA-seq data, we chose three miRNAs (miR-320a-5p, miR-486-5p, and miR-122-5p) for validation of their expression by qRT-PCR in 26 samples between the mild and the severe dengue patients. Seven OFI samples, negative for dengue NS1-IgM or IgG, were used as the negative control. While miR-122-5p remained undetectable in all the OFI patients, it was present in 100% of the DI and DS patients (Fig. 3), whereas miR-320a-5p and miR-486-5p were detected in all the sample groups and were significantly upregulated in the DS group compared to the DI group (Fig. 3).

**FIG 4:**
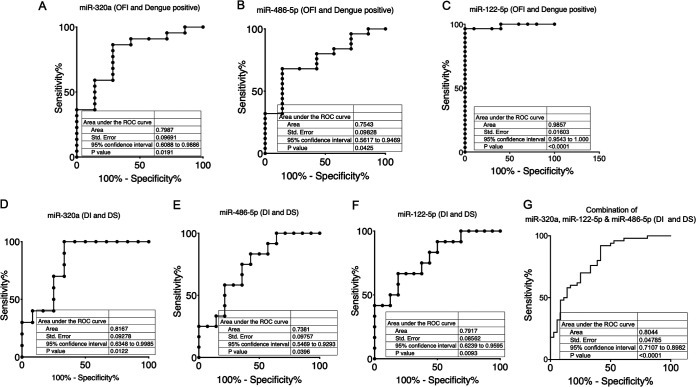
Receiver-operating characteristic (ROC) curve analysis. ROC curve analysis was carried out for differentiating between OFI and dengue infection or within the DI and DS groups of dengue patients. Sensitivity is plotted as a function of specificity for the different miRNAs. The area under the curve (AUC) is a measure of how well a quantitative test can distinguish between the two groups. (A to C) ROC for OFI (*n* = 7) versus all dengue patients (*n* = 26) is plotted. (D to G) ROC for DI (*n* = 14) versus DS (*n* = 12) groups for each of the three miRNAs is plotted either individually or together in the plasma of dengue patients.

Next, we plotted the receiver-operating characteristic (ROC) curves to analyze the sensitivity and specificity of these miRNAs and to determine if any of these were associated with the dengue disease progression. An area of 1 indicates that the miRNA can differentiate the two groups, whereas an area of 0.5 indicates that it fails to distinguish the two groups. We first compared the OFI group with the dengue (all patients) group and found that the areas under the ROC curve (AUC) for miR-320a-5p, miR-486-5p, and miR-122-5p were 0.79, 0.75, and 0.983, respectively (Fig. 4A to C), suggesting that the levels of miR-320a-5p and miR-486-5p can distinguish between the dengue-positive patients and control OFI with moderately high sensitivity and specificity. Within the dengue-positive groups, we next compared the DI and DS groups. The AUCs for miR-320a-5p, miR-486-5p, and miR-122-5p were 0.81, 0.73, and 0.79, respectively, suggesting that the levels of these miRNAs can distinguish between the DI and DS cases with moderately high sensitivity and specificity (Fig. 4D to F). Further, to understand if combined miRNA detection provided a better diagnostic power, the ROC analysis was performed for the three miRNAs together as described by Liu et al. (19). Our analysis suggested that the combination of the miRNAs increased the AUC, 95% confidence interval (CI) value, and statistical significance as shown in Fig. 4G.

### Validation of differentially expressed miRNAs in DENV-infected cultured cells and PBMCs from dengue patients.

**FIG 5:**
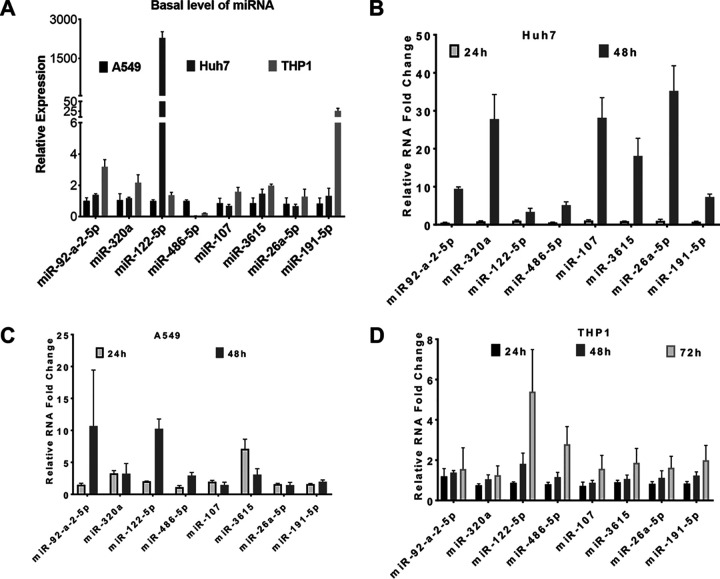
Validation of differentially expressed miRNAs in cultured cells infected with DENV. Huh7 and A549 cells were mock infected or infected with DENV (MOI 5) and harvested at 24 h and 48 h p.i. Similarly, THP1 cells infected with DENV (MOI 3) were harvested at 24 h, 48 h, and 72 h p.i. The total RNA from the cells was isolated, and relative levels of viral RNA and miRNAs were determined by qRT-PCR. The U6 transcripts were used for normalization. (A) The relative abundance of different miRNAs in uninfected cells was measured against the expression of the respective miRNA in A549 cells. (B to D) Relative expression of different miRNAs in DENV-infected cells at different time points. The bar graph represents the data with mean and standard deviation.

Circulating miRNAs in the plasma reflect the miRNAs that are released from the cells. To understand this further, we studied the expression of the various miRNAs that were either significantly upregulated (miR-92-a-2-5p, miR-122-5p, miR-107-5p, miR-3615-5p, miR-320a-5p, and miR-486-5p) or downregulated (miR-26a-5p and miR-191-5p) in the plasma of the DS patients in the cultured cells (Huh7, A549, and THP1) infected with DENV. We observed a significant variation in the relative abundance of the eight selected miRNAs in these cells at the basal level (Fig. 5A). Although all these miRNAs were upregulated in response to DENV infection, their upregulation patterns were different. The basal level of miR-486-5p in A549 cells was higher than that in Huh7 and THP1 cells. A relatively high abundance of miR-92a-2-5p, miR-320a-5p, and miR-191-5p was observed in THP1 cells compared to that in A549 and Huh7 cells. However, miR-107-5p, miR-26a-5p, and miR-3615-5p were detectable in all three cell lines with comparable levels. Importantly, miR-122 levels were exclusively enriched in Huh7 cells but were detectable in both A549 and THP1 cells. In Huh7 cells, all these miRNAs were upregulated in response to DENV infection at 48 h postinfection (p.i.), whereas miR-320a-5p and miR-3615-5p levels increased by 24 h p.i. in A549 cells. No significant changes were observed in the expression of miR-26a-5p and miR-191-5p at 24 and 48 h p.i. in these cells, whereas miR-92a, miR-122, and miR-486 were upregulated only at 48 h p.i. (Fig. 5B and C). Interestingly, a late response was seen in THP1 cells, where the upregulation of these miRNAs was observed only at 72 h p.i. (Fig. 5D).

**FIG 6:**
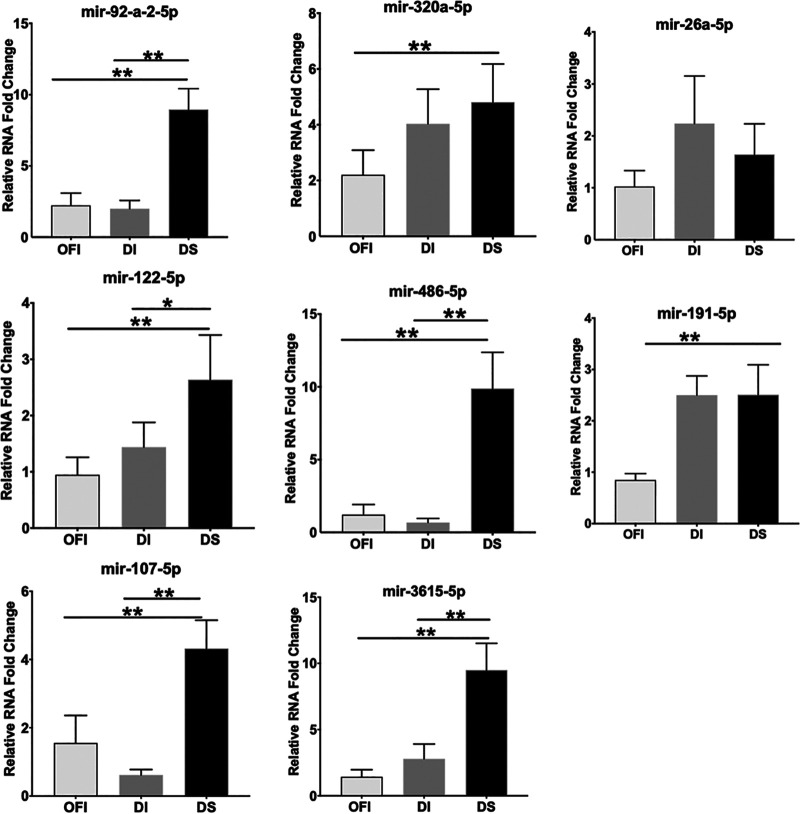
Validation of differentially expressed miRNAs in the PBMCs of the patients. Total RNA was isolated from the PBMCs of six patients each in the OFI, DI, and DS groups, and miRNA expression was studied by qRT-PCR using U6 as the endogenous reference gene. The relative expression of the miRNAs in the DI and DS groups was determined with respect to that seen for the respective miRNA in the OFI group. The bar graph shows the mean and standard deviation of the relative expression of the indicated miRNA.

To validate that these differences in miRNA expression are associated with disease progression, we studied the miRNA expression in peripheral blood mononuclear cells (PBMCs) isolated from patients in the dengue-negative OFI group and those in the DI and DS groups. Compared to the OFI and DI groups, the expression of miR-92-a-2-5p, miR-122-5p, miR-107-5p, miR-3615-5p, and miR-486-5p was significantly enhanced in the DS group (Fig. 6), validating the observation made for these miRNAs in the plasma samples of the DS group patients. However, no significant increase in the expression of miR-320a-5p, miR-26a-5p, and miR-191-5p was seen in the PBMCs between the DI and DS groups (Fig. 6).

### Biological functions that may be affected by the dysregulated miRNAs.

**FIG 7:**
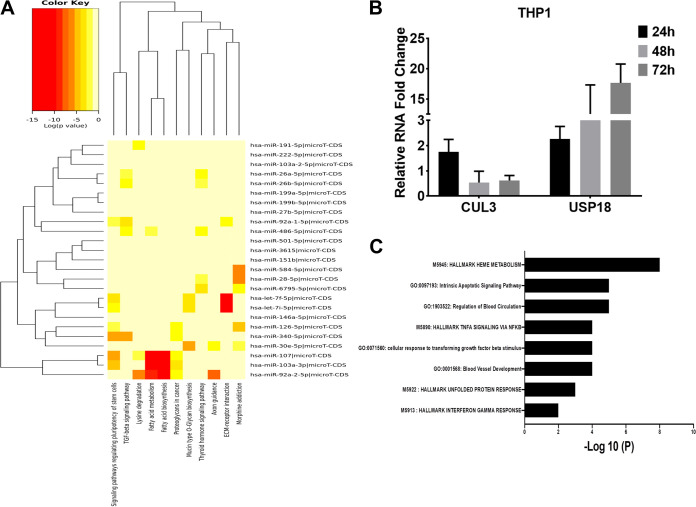
Dysregulated miRNAs affecting the global metabolic pathways and validation of target genes. The miRNA target gene and pathway enrichment analyses were performed using miRPath v.3. (A) Heatmap represents miRNAs and targeted pathways. Clustering is based on significance levels. Darker colors represent a higher significance. On the vertical axis, miRNAs displaying similar pathway targeting patterns are clustered together. (B) Determining the expression of CUL3 and USP18 in DENV-infected cell lines. THP1 cells, infected at a multiplicity of infection (MOI) of 3, were harvested at 24 h, 48 h, and 72 h p.i. The total RNA from cells was isolated, and relative levels of viral RNA, CUL3, and USP18 genes were determined by qPCR. (C) Pathway enrichment analysis of genes dysregulated in PBMCs of dengue-infected patients and targeted by the miRNAs. Pathways are plotted against the *P* values.

The DIANA-miRPath v3.0 web server was used to perform the *in silico* pathway analysis to understand the potential biological functions affected by the 89 dysregulated miRNAs in the dengue patients. Signaling pathways regulating the pluripotency of stem cells, transforming growth factor beta (TGF-β) signaling pathways, fatty acid biosynthesis and metabolism, and extracellular matrix (ECM) receptor interaction were the top 4 significantly targeted pathways (Fig. 7A). From this analysis, it is also evident that fatty acid biosynthesis and metabolism, as well as ECM receptor interaction pathways, were among the most statistically significant targeted pathways and let-7f-5p, let-7i-5p, miR-107-5p, miR-103, and miR-92a-2-5p were closely related to miRNAs that can target these pathways.

### Genes in the PBMCs of the dengue patients targeted by dysregulated microRNAs.

The online tools miRSystem and DIANA-miRPath v3.0 were employed to investigate the target genes of the dysregulated miRNAs identified in the study. The integrated list generated, consisting of 6,416 genes with the validated status or a minimum of three hits from miRSystem, had 89 commonly dysregulated miRNAs in all dengue patients. We compared the predicted target 6,416 genes against the 336 genes significantly altered in the PBMCs of the dengue patients in our previous study (20). A total of 77 of the previously reported dengue infection-associated genes (20) were found to be targeted by these differentially expressed miRNAs (Table S3). Further narrowing identified 12 genes that were significantly altered in the DI, DWS, and DS groups, and their expressions were negatively correlated with the expression of miRNAs, suggesting a potential target for these miRNAs (Table S4). Several upregulated genes (e.g., MPO, OLFM4, NAMPT, and CACNA1E) that were associated with the neutrophil activation process were found to be targeted by the several miRNAs whose expressions were downregulated in the DS group of patients compared to the DI group. Apart from this, two important downregulated genes, CUL3 and USP18, were identified as the target for several miRNAs. These two important genes play a vital role in DENV replication and antiviral response. In our previous study (20), we observed a significant decrease in the CUL3 transcripts in the PBMCs of the DS patients. In DENV-infected THP1 cells, CUL3 levels decreased significantly in a time-dependent manner (Fig. 7B). CUL3 may be targeted by miR-92a-5p and miR-486-5p, whose expression was upregulated in DENV-infected THP1 cells (Fig. 5) as well as in the PBMCs of the DS patients (Fig. 6). Importantly, USP18 levels were upregulated in the DENV-infected THP1 cells (Fig. 7B). The USP18 gene encodes a type 1 interferon (IFN)-stimulated gene, which is a negative regulator of type 1 IFN signaling. Our previous RNA-seq data showed a significant upregulation of USP18 in the dengue patients and are inversely correlated with the reduced levels of miR-191-5p that can target USP18 (Table S4). However, levels of miR-191-5p did not change in PBMCs between the DI and DS patients and were slightly increased in THP1 cells at 72 h p.i. (Fig. 5).

10.1128/mSystems.00724-20.4 TABLE S3 List of 77 genes and their expression values in PBMCs of dengue patients. Download Table S3, DOCX file, 0.02 MB. Copyright © 2020 Saini et al. 2020 Saini et al. This content is distributed under the terms of the Creative Commons Attribution 4.0 International license.

10.1128/mSystems.00724-20.5 TABLE S4 Inverse expression of circulating microRNAs and their target genes in PBMCs. Download Table S4, DOCX file, 0.02 MB. Copyright © 2020 Saini et al. 2020 Saini et al. This content is distributed under the terms of the Creative Commons Attribution 4.0 International license.

To understand the pathways that are affected due to the modulation of microRNAs in the PBMCs, we carried out the pathway enrichment analysis for genes targeted by the 89 common dysregulated microRNAs in the plasma of the DWS and DS patients. Interestingly, 77 genes were associated with pathways that were possibly modulated by the dysregulated miRNAs and associated with dengue disease progression. Among them, three pathways, e.g., hallmark of heme metabolism, hallmark of tumor necrosis factor alpha (TNF-α) signaling, and cellular response to transforming growth factor-β stimulus, were found to be targeted by the microRNAs enriched in all categories of the dengue patients (Fig. 7C). The target genes in these pathways probably modulated by the miRNAs are listed in Table 3.

**TABLE 3:** List of genes dysregulated in the PBMCs of dengue patients and associated pathways

Description	−Log_10_(P)	Hits
M5913: hallmark interferon gamma response	2.4	IRF4|PTGS2|NAMPT|USP18
M5922: hallmark unfolded protein response	3.3	SLC1A4|WFS1|XBP1|CHAC1
GO:0001568: blood vessel development	4	CAV1|COL1A1|COL1A2|EGR1|EPHB2|SMAD6|MCAM|PTGS2|XBP1
GO:0071560: cellular response to transforming growth factor beta stimulus	4.2	CAV1|COL1A1|COL1A2|SMAD6|PMEPA1|CGN
M5890: hallmark TNFA signaling via NFKB	4.3	HBEGF|EGR1|MXD1|PTGS2|NAMPT|PMEPA1
GO:1903522: regulation of blood circulation	4.5	ADRA2B|ASPH|CAV1|HBEGF|JUP|PTGS2|THRB
GO:0097193: intrinsic apoptotic signaling pathway	4.5	BCL2L1|CAV1|E2F2|WFS1|XBP1|CUL3|CHAC1
M5945: hallmark heme metabolism	7.7	ANK1|E2F2|MXI1|SLC2A1|SLC6A9|TMCC2|FAM46C|RBM38|RANBP10

## DISCUSSION

Circulating microRNAs play an important role in disease manifestation, progression, pathology, and the disease outcome. In the present study, we characterized the landscape of circulating miRNAs in the plasma of the dengue patients with or without severe symptoms, validated the findings in the DENV-infected tissue-cultured cells, and through the *in silico* analysis predicted the role of specific circulating miRNAs in modulating the important metabolic pathways in the cell. Previously, profiling of dysregulated miRNAs had been done using the miRNA PCR arrays in the serum of the DENV-infected patients (21, 22). The present study is more comprehensive as the small RNA sequencing technique was used to cover all the microRNAs reported in the miRbase database, whereas the previous study used a PCR-based miRNA array that covered only 752 miRNAs. Moreover, the present study used different categories of dengue patients based on disease severity that provided a basis for identifying the miRNAs associated with the dengue disease progression. In addition, our study included the OFI group as an important control to provide information relevant to dengue fever only. We observed that 17 miRNAs were common between the two studies. However, only six miRNAs were differentially expressed in different categories of dengue patients. To the best of our knowledge, this is the first comprehensive, small RNA sequencing-based study providing a global view of the circulating miRNAs in the plasma of patients infected with DENV.

Our sequencing data showed a distinct miRNA spectrum in the plasma of different categories of dengue patients. Using the *in silico* analysis, 25 miRNAs were found to be differentially expressed between the DI and DS patients. Eight of the topmost dysregulated miRNAs (miR-92a-2-5p, miR-320a-5p, miR-191-5p, miR-107-5p, miR-26a-5p, miR-3615-5p, miR-122-5p, and miR-486-5p) were further validated in three different cell lines following the DENV infection. Interestingly, the relative abundance of these miRNAs at the basal level was different in different cells. However, as the viral load increased, the relative expression of these miRNAs also increased. To test that this miRNA upregulation was DENV specific, we studied their expression in THP1 cells infected with the Japanese encephalitis virus (strain P20778), which also belongs to the same *Flaviviridae* family of viruses as does DENV. Except for miR-92a-2-5p, none of the other seven miRNAs were upregulated at 72 h p.i. (data not shown), suggesting that the dysregulation of these miRNAs was DENV specific and may have implications for the development of severity in the dengue patients.

The miRNA miR-486-5p is highly abundant in the peripheral blood and plasma, is specifically upregulated in the erythroid lineage, and regulates the normal erythropoiesis (23–25). Erythropoiesis suppression occurred mainly during the dengue fever phase, and erythropoiesis started to be restored in the critical phase (26). An increased level of miR-486-5p in circulation in the severe dengue cases thus supports this observation. Besides, miR-486-5p also plays an antiviral role against the influenza virus (27). This miRNA was found to target a viral gene segment of the influenza virus and attenuate its replication. Similarly, a bioinformatics study identified two potential binding sites of miR-486-5p on DENV serotype 1 (DENV-1) and 3 genomes but did not find any binding sites on DENV serotypes 2 and 4 (28). In this study, we used DENV serotype 2 and did not observe any negative correlation between the intracellular DENV genomic RNA level and miR-486-5p expression in different cell lines infected with DENV (data not shown). Further study is needed to validate the *in silico* prediction of the antiviral role of miR-486-5p against DENV serotypes 1 and 3. The upregulation of miR-486-5p in DENV-infected THP1 cells probably reflects its association with the host inflammatory response. This is based on the observation that miR-486-5p was upregulated in the serum of sepsis patients (29). Notably, the expression of miR-486-5p and inflammatory responses were elevated in the lipopolysaccharide (LPS)-stimulated macrophages, and miR-486 silencing alleviated the inflammatory response (30). Thus, miR-486-5p is an important miRNA needing to be studied in great detail in dengue infection to understand its role in the disease manifestation.

Another miRNA, miR-320a, upregulated in this study, is also highly abundant in the blood exosomes and fibroblasts (31). Interestingly, miR-320a is found to be secreted by activated neutrophils (31). Neutrophil activation leading to neutrophil extracellular trap (NET) formation is an important event in dengue infection, probably associated with dengue disease progression. A higher level of circulating exosomes containing miR-320a highlights neutrophil activation. The miR-320a levels increased in the PBMCs of dengue patients with severe disease manifestation. Several genes, e.g., NAMPT and CACNA1E, which were identified as the potential target genes of miR-320a *in silico*, were significantly downregulated under the severe dengue conditions. These genes are involved in the neutrophil maturation and activation process (32).

Here, we also studied the plasma samples from nine patients followed up at two different time points. We identified five miRNAs (miR-486, miR-92a, miR-320a, miR-191-5p, and miR-378a-5p), which were found in both the DWS and DS groups and had negative expression patterns between the severe and recovered patients. To establish their potential as dengue disease progression markers, their validation in a larger number of plasma samples of different categories of dengue patients is warranted. Interestingly, miR-122-5p, which is highly abundant in hepatocytes, was found to be significantly enriched in the plasma of the DS patients. Hepatic dysfunction is a well-reported feature in both DF and DHF (33). The finding of a higher level of miR-122-5p in the DS patients supports the previous studies where the cases with acute dengue infection showed liver dysfunction and often resulted in acute liver failure with fatal outcomes (34–36). Similarly to our observations, Tambyah et al. (37) also observed a huge upregulation of miR-122-5p levels in the blood of the dengue severe patients. It was also of great importance that miR-122-5p was altogether undetectable in the OFI group of patients while it was significantly upregulated in all dengue patients. The ROC analysis indicated that its presence could be used to distinguish dengue patients with a high level of confidence. Further validation of miR-122-5p as a potential biomarker of dengue infection is desirable.

To our knowledge, this is the first effort to understand the level of the circulating miRNAs in different disease stages of dengue patients. A total of 39 patients’ plasma was profiled separately as a part of the discovery cohort to get comprehensive information on the circulating miRNAs. However, there are some limitations to our study. First, due to the small sample size in the validation cohort, the results presented may be considered an exploratory study. Validation of the results in a larger cohort is required for a higher level of confidence. Second, the study we conducted had only the teenaged to the middle-aged group of patients. The differential expression of the miRNAs should be studied in an extended cohort with a broader age range of patients. For recruiting the participants from diverse populations, the formation of a dengue biobank through collaborations would be a very useful resource to validate the miRNAs identified in our study. In our study population, the predominant virus was DENV serotype 2, although DENV serotypes 1 and 3 were also there in a minor proportion. India being a vast country and all four DENV serotypes circulating across the country, the plasma samples from the patients infected with different serotypes may need to be studied for a better understanding of the serotype-specific profile of circulating miRNAs. Because we studied in a cross-sectional design the dengue patients who came to the outpatient clinics, we do not know if the miRNA changes in the dengue patients could serve as risk factors or could be predictive of the dengue illness. To understand this, it is important to measure the abundances of these miRNAs during the development of the dengue illness in a longitudinal study.

In summary, our results suggest that some of the miRNAs differentially expressed during the different stages of dengue infection have the potential to serve as a marker for dengue disease progression. While the viral and serum proteins, and the other biochemical and molecular parameters, have the diagnostic power (38–40), an additional predictive and classification accuracy is expected by integrating the information on some of the circulating miRNAs identified in this study.

## MATERIALS AND METHODS

### Study population.

The study was conducted according to the ethical standards of the Declaration of Helsinki. It was approved by the Ethics Committee of School of Tropical Medicine (STM) Kolkata and Guru Teg Bahadur (GTB) Hospital, Delhi. All these samples were collected during 2014 to 2016, as described earlier (20). Written consent was obtained from each participant. A total of 65 dengue patients and 7 other febrile illness (OFI) controls were included in this study. The demographic, clinical, and virological characteristics were shown in Table 1. The discovery cohort comprised 39 dengue NS1-IgM-positive patients who visited the outpatient clinic of the STM, Kolkata, and GTB Hospital, Delhi, for the treatment of febrile illness. Out of 39 patients, nine patients were followed up at two time points (day 0 and day 5). Day 0 is considered the day of enrollment in the outpatient clinics. Twenty-six patients’ plasma samples including those from 14 mild and 12 severe dengue patients were used for the validation study. Once patients were enrolled, blood was collected, dispensed, and mixed gently into an EDTA collection tube to ensure proper exposure to the EDTA-coated walls. The plasma was isolated from blood by centrifugation at 2,000 relative centrifugal force (rcf) for 15 min at room temperature. The clear top layer was transferred to the RNase-free tubes and stored at −80°C.

The blood samples were used to check the platelet counts, NS1-IgM, and the DENV serotype. Patients were categorized per the WHO (2009) criteria as patients with uncomplicated dengue infection (DI; *n* = 9), dengue with a warning sign (DWS; *n* = 14), and dengue severe (DS; *n* = 16). The mean age, fever duration, DENV serotypes, and the platelet count of the patients are mentioned in Table 1. In addition, nine patients were further followed up and blood samples were collected on day 5 from the first reporting date and included in the sequencing studies. Among the 9 samples, 5 exhibited lower platelet counts at day 0, and an increase in platelet count was observed at day 5. The rest exhibited higher platelet counts at day 0, and a decrease in platelet count was observed at day 5. The other febrile illness (OFI) patients came to hospitals with flu-like symptoms; however, they were serology negative for dengue NS1-IgM and IgG and also PCR negative for dengue RNA.

### Cell culture and DENV infection.

Human lung carcinoma cells (A549; ATCC) and human hepatocellular carcinoma cells (Huh7; ATCC) were cultured in Dulbecco’s modified Eagle medium (HiMedia), human monocytic cells (THP1; ATCC) were cultured in RPMI 1640 medium (HiMedia), African green monkey kidney cells (Vero; ATCC) were cultured in minimum essential medium (HiMedia), and Aedes albopictus mosquito cells (C6/36; ATCC) were cultured in L-15 medium (HiMedia) with 10% fetal bovine serum (FBS) (HyClone; SH30070). All media were additionally supplemented with 100 μg/ml penicillin-streptomycin and 2 mM l-glutamine.

To perform the *in vitro* experiments, A549 and Huh7 cells were infected by DENV serotype 2 at a multiplicity of infection (MOI) of 5, and THP1 cells were infected at an MOI of 3 along with mock-infected control. Experiments were done in triplicates. The cells were incubated in the serum-free medium when inoculated with the virus for 2 h; the medium was then replaced with a 2% FBS-containing medium.

### DENV propagation.

Dengue virus serotype 2 (DENV-2) (IND/P23085/1960 strain, GenBank accession no. JQ922552.1) was used in this study. Briefly, the virus propagation was done by infecting 70 to 80% confluent Aedes albopictus C6/36 cells (CRL-1660; ATCC) in serum-free medium at an MOI of 0.2, and the cells were thereafter incubated in L-15 medium with 2% FBS for 5 days. The supernatant was then harvested and concentrated 10 times. The virus was aliquoted and stored at −80°C. The titer of the virus was determined by performing the focus formation assay on Vero cells, as described previously (41).

### RNA isolation and sequencing.

The microRNAs were isolated from 150 μl of the plasma according to the miRNeasy RNA isolation protocol (Qiagen). The quality of the isolated small RNA was checked using the small RNA chip in the 2100 Bioanalyzer (Agilent) for each sample, and the quantitation was performed in a Qubit fluorometer using a microRNA assay kit (Invitrogen). The small RNA sequencing library preparation was performed using the Illumina TruSeq small RNA library prep kit according to the manufacturer’s instructions. Four nanograms of small RNA per sample was used for the library preparation. Adapters were ligated to each of the 3′ and 5′ ends of the RNA fragments and then reverse transcribed and amplified to generate the cDNA library. A gel purification step that selected bands between 145 and 160 bp of the cDNA library was performed to prepare the final small RNA sequencing library for clustering and sequencing. The quality of the small RNA-seq libraries was checked using the high-sensitivity chips in the 2100 Bioanalyzer (Agilent), and the final library quantification was performed in a Qubit fluorometer. Single-read 1 × 50-bp sequencing of these libraries was performed in HiSeq-2500 (Illumina).

### Sequence analysis.

The quality check of the sequences was done by FastQC. There were 9 samples in the DI group, 14 samples in the DWS group, and 15 samples in the DS group. For each sample, the two lane FastQC files were merged into one for further processing. Adapter sequences were trimmed from the reads using cutadapt v. 1.2.1, discarding reads with length less than 18 nucleotides and more than 27 nucleotides.

### Identification of miRNAs and analysis of differential expression.

The miRNA sequence reads were analyzed using the miRDeep2 software (42) using a Linux platform to generate the basic miRNA expression reads. For the mapping of the reads to the human genome hg19, MiRDeep2 used Bowtie. We have considered the miRNAs which were expressed in at least two-thirds of the samples in each group. The RNA sequencing and data analysis pipeline are shown in Fig. 1.

### Validation of miRNA expression by quantitative reverse transcription-PCR (qRT-PCR).

The expression levels of the candidate miRNAs selected from the RNA-seq analysis were validated with the use of miRNA qRT-PCR. Total RNA (7 μl) purified from the individual plasma sample was subjected to reverse transcription using the miRNA-specific stem-loop primers and the miRNA reverse transcription kit (Qiagen) following the manufacturer’s protocol. The real-time PCR assay was performed in a 5-μl reaction mixture that contained 2 μl 2× Sybr green fast reaction, 2.5 μl diluted cDNA (1:15), and 0.5 μl miRNA assay primers (Qiagen). The thermal cycling procedure was set as follows: an initial denaturation step at 95°C for 10 min, 40 cycles of PCR amplification at 95°C for 15 s, and 60°C for 1 min. Each RNA sample was run in duplicate. The expression level of each miRNA was individually normalized to endogenous miR-423-5p.

### Functional association of the miRNA target gene.

The miRNA pathway analysis and the hierarchical clustering of targeted pathways and miRNAs were performed using the open-access webserver DIANA-miRPath v3.0 inbuilt with DIANA-micro T-CDS (43, 44), a broadly used miRNA target prediction algorithm exhibiting the highest sensitivity and specificity compared with other analyses. The hierarchical clustering analysis of targeted pathways and miRNAs was also performed using the inbuilt DIANA-miRPath v3.0 software. Additionally, DEMis were submitted for “miRNAs to Target Genes” analysis on miRSystem16, using the default settings. DEMis which were not found in the miRSystem database were checked individually on DIANA-miRPath to identify the evidence-based target genes. The identification of overlaps between the individual target lists was made, and it was then compared with the PBMC transcriptome data (45). Overlaps between the individual target lists were sorted. For identifying the biological processes of the miRNA target genes, functional enrichment analyses of pathways were performed using the default Metascape setting (46).

### Statistical analysis.

Raw amplification data of the real-time PCR were expressed as cycle threshold (*C_T_*) values. The mean *C_T_* was calculated from the duplicates of each sample and each miRNA amplification. The median of the normalizer *C_T_* values (*C_T_*-miR-423-5p) from all samples was calculated as described previously (5). Statistical analysis of *C_T_* and normalized *C_T_* (n*C_T_*) was performed using GraphPad Prism 7. microRNA levels were compared between patients with dengue and OFI controls using the Mann-Whitney U test. The correlation analysis was performed using the Spearman test. A *P* value of <0.05 was considered significant.

### Data availability.

All the small RNA-seq data generated in this study have been submitted to GEO under accession number GSE150623.
