# Supplementary material for: High-Throughput RNA Sequencing Analysis of Plasma Samples Reveals Circulating microRNA Signatures with Biomarker Potential in Dengue Disease Progression
Source: mSystems. 2020 Sep 15;5(5):e00724-20. doi: 10.1128/mSystems.00724-20 (PMC7498686; doi:10.1128/mSystems.00724-20)
Supplement: TABLE S1 [file mSystems.00724-20-st001.docx]

Table S1. DS group-specific differentially-expressed miRNAs.

| miRNA | *^a^*Log2FC | *^b^*logCPM | p-value | *^c^*FDR |
| --- | --- | --- | --- | --- |
| hsa-miR-6499-5p_MIMAT0025450 | 4.379861 | 7.825349 | 3.82E-08 | 1.13E-06 |
| hsa-miR-122-5p_MIMAT0000421 | 4.310399 | 8.461546 | 5.42E-05 | 0.000402 |
| hsa-miR-193b-5p_MIMAT0004767 | 2.437802 | 7.600976 | 0.01441 | 0.036315 |
| hsa-miR-320c_MIMAT0005793 | 1.751987 | 7.963905 | 0.003829 | 0.015492 |
| hsa-miR-320a_MIMAT0000510 | 1.678662 | 13.99822 | 0.000836 | 0.004376 |
| hsa-miR-92b-5p_MIMAT0004792 | 1.332492 | 10.9752 | 0.009852 | 0.029628 |
| hsa-miR-21-5p_MIMAT0000076 | -0.72826 | 13.22186 | 0.016344 | 0.039315 |
| hsa-miR-181a-5p_MIMAT0000256 | -0.76274 | 12.78285 | 0.011224 | 0.030271 |
| hsa-miR-27b-5p_MIMAT0004588 | -0.95394 | 12.22436 | 0.001405 | 0.00658 |
| hsa-miR-30e-5p_MIMAT0000692 | -0.97078 | 11.12559 | 0.004183 | 0.015513 |
| hsa-miR-30d-5p_MIMAT0000245 | -0.97117 | 12.26875 | 0.00312 | 0.013221 |
| hsa-miR-151b_MIMAT0010214 | -1.59063 | 12.04632 | 1.07E-05 | 9.80E-05 |
| hsa-miR-127-5p_MIMAT0004604 | -2.60388 | 9.006513 | 3.50E-05 | 0.000283 |

*^a^* Log Fold Change; *^b^* Log Counts Per Million; *^c^* False Discovery Rate
