# Supplementary material for: High-Throughput RNA Sequencing Analysis of Plasma Samples Reveals Circulating microRNA Signatures with Biomarker Potential in Dengue Disease Progression
Source: mSystems. 2020 Sep 15;5(5):e00724-20. doi: 10.1128/mSystems.00724-20 (PMC7498686; doi:10.1128/mSystems.00724-20)
Supplement: TABLE S2 [file mSystems.00724-20-st002.docx]

Table S2. List of miRNAs identified in follow-up patients and their expression inversely correlated with expression observed in DWS and DS patients

|  | MicroRNA | Follow up  (Low to High Platelet count)  Log2 Fold change | DI vs DWS  Log2 Fold change | DI vs DS  Log2 Fold change |
| --- | --- | --- | --- | --- |
| 1 | hsa-let-7i-5p_MIMAT0000415 | 0.8721162 | -1.6067182 | -1.336089 |
| 2 | hsa-miR-146a-5p_MIMAT0000449 | 1.10351929 | -1.2675069 | -1.4015334 |
| 3 | hsa-miR-151b_MIMAT0010214 | 1.41408312 | 0.80654341 | -1.5906306 |
| 4 | hsa-miR-191-5p_MIMAT0000440 | 1.57559367 | -1.8295633 | -2.5879465 |
| 5 | hsa-miR-22-5p_MIMAT0004495 | 0.57467475 | 0.83541765 | -0.7488909 |
| 6 | hsa-miR-222-5p_MIMAT0004569 | 0.68257262 | -2.3012619 | -1.9595756 |
| 7 | hsa-miR-26a-5p_MIMAT0000082 | 1.18947173 | -2.9534094 | -3.1114024 |
| 8 | hsa-miR-26b-5p_MIMAT0000083 | 0.83572102 | -1.7647747 | -1.6626788 |
| 9 | hsa-miR-28-5p_MIMAT0000085 | 0.8373813 | -0.6713336 | -1.309785 |
| 10 | hsa-miR-320a_MIMAT0000510 | 0.8433801 | 1.0975632 | 1.67866243 |
| 11 | hsa-miR-320b_MIMAT0005792 | 0.82647289 | 1.04576795 | 1.46663373 |
| 12 | hsa-miR-3615_MIMAT0017994 | 0.69225782 | 1.96027445 | 1.90729004 |
| 13 | hsa-miR-378a-5p_MIMAT0000731 | 1.17272698 | 2.30423216 | 2.59451 |
| 14 | hsa-miR-486-5p_MIMAT0002177 | 0.57422595 | 3.4399157 | 2.25284745 |
| 15 | hsa-miR-584-5p_MIMAT0003249 | 1.18084501 | -1.7761045 | -1.4897141 |
| 16 | hsa-miR-92a-1-5p_MIMAT0004507 | 0.85617592 | 1.96558522 | 1.74442041 |
| 17 | hsa-miR-92a-2-5p_MIMAT0004508 | 0.84143496 | 1.80509774 | 1.56694336 |
|  |  |  |  |  |
| **miRNAs detected both follow-up (High to low platelet count) and DI vs DWS** | | | | |
|  | MicroRNA | **high to low** | | **DI to DWS** |
|  | hsa-miR-10b-5p_MIMAT0000254 | 0.654813544 | | 1.619375658 |
|  | hsa-miR-1307-5p_MIMAT0022727 | 0.607824136 | | 1.010444037 |
|  | hsa-miR-409-5p_MIMAT0001638 | -0.878300023 | | -1.763358186 |
|  | hsa-miR-363-5p_MIMAT0003385 | 0.58671022 | | 1.75658525 |
