# Supplementary figures and images for: High-Throughput RNA Sequencing Analysis of Plasma Samples Reveals Circulating microRNA Signatures with Biomarker Potential in Dengue Disease Progression
Source: mSystems. 2020 Sep 15;5(5):e00724-20. doi: 10.1128/mSystems.00724-20 (PMC7498686; doi:10.1128/mSystems.00724-20)

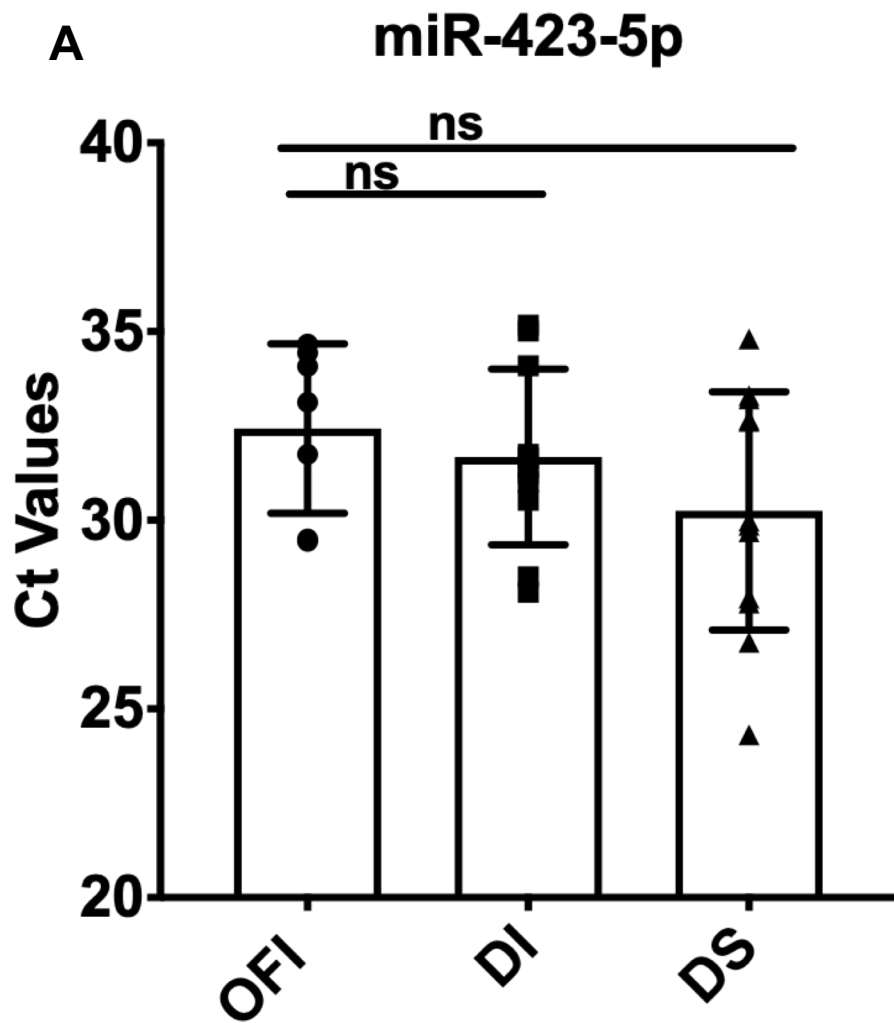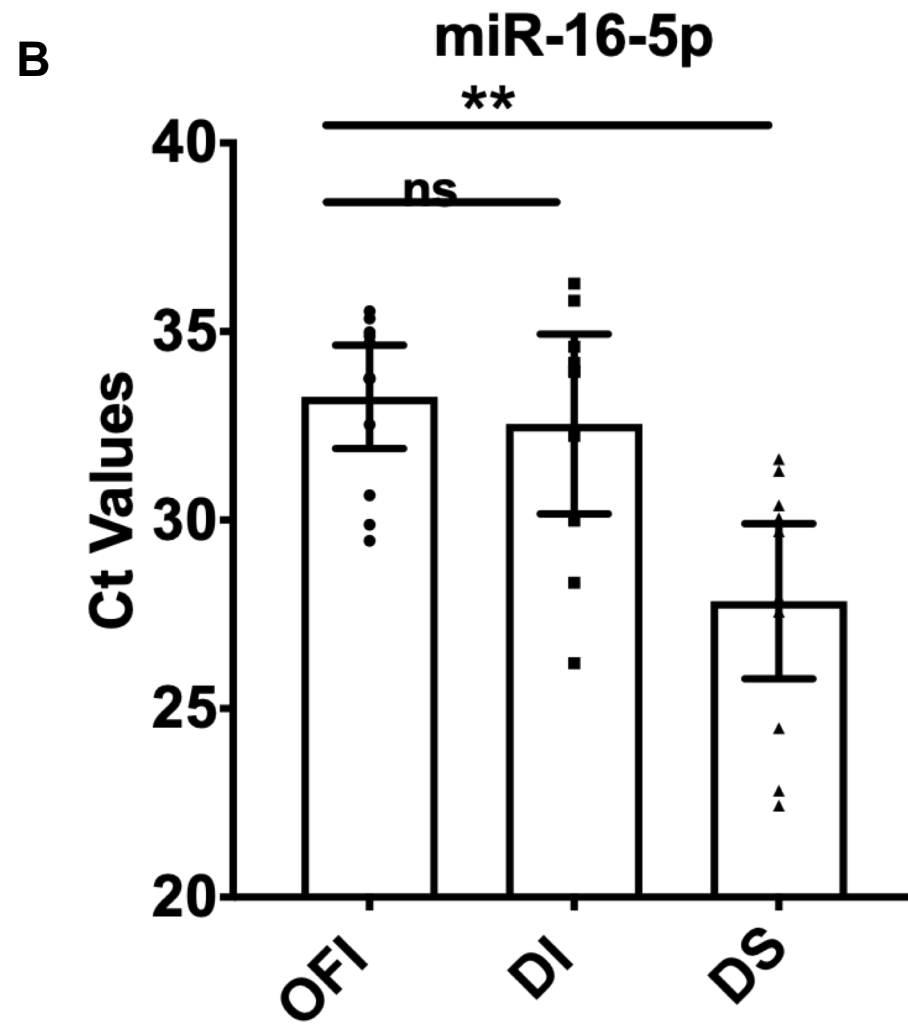

Supplement: FIG S1 [file mSystems.00724-20-sf001.pdf]
