# Supplementary material for: High-Throughput RNA Sequencing Analysis of Plasma Samples Reveals Circulating microRNA Signatures with Biomarker Potential in Dengue Disease Progression
Source: mSystems. 2020 Sep 15;5(5):e00724-20. doi: 10.1128/mSystems.00724-20 (PMC7498686; doi:10.1128/mSystems.00724-20)
Supplement: TABLE S3 [file mSystems.00724-20-st003.docx]

Table S3. List of 77 genes and their expression values in PBMCs of dengue patients

| Genes | *^a^* LFC_DI | LFC_DWS | LFC_DS |
| --- | --- | --- | --- |
| COL1A2 | 0.944061 | 3.51079 | 5.06993 |
| COL1A1 | 1.70202 | 1.18668 | 5.55996 |
| ADRA2B | -1.74042 | -2.75418 | -5.32209 |
| MYBL2 | -0.226665 | 2.52839 | 2.27664 |
| GLDC | -1.36706 | 2.29881 | 1.91779 |
| FNDC3B | -0.579095 | -1.70289 | -2.3544 |
| SLC16A14 | -1.29723 | 3.19528 | 2.53924 |
| SMAD6 | 1.47654 | 2.68488 | 2.21142 |
| COL9A3 | 0.944397 | 2.74903 | 2.55891 |
| MXI1 | 1.34562 | 1.00664 | 2.63957 |
| RBM38 | 1.09609 | NA | 2.54815 |
| TRPS1 | -1.24822 | -3.65576 | -2.78752 |
| CBLB | -1.42407 | -2.20176 | -1.09007 |
| NAALADL2 | -0.390845 | -2.4036 | -1.20045 |
| CCNF | 0.071639 | 2.2659 | 1.61801 |
| BCL2L1 | 1.39445 | 1.17364 | 2.92443 |
| SRGAP1 | -0.47582 | -2.70886 | -1.97833 |
| CAV1 | -0.923146 | 2.27135 | 2.14961 |
| CACNA1E | 0.366446 | -2.64506 | -2.6054 |
| ZNF215 | -0.7152 | 2.09483 | NA |
| RANBP10 | 0.818491 | 0.952266 | 2.05273 |
| SLC6A9 | 2.08454 | 3.50399 | 4.29697 |
| MXD1 | 0.124053 | -2.29532 | -2.70366 |
| FAM171A1 | 0.382368 | 2.29986 | 1.95861 |
| BCL7A | -0.061499 | 2.00328 | 1.63595 |
| E2F2 | 1.08626 | 2.30844 | 2.79812 |
| FAM46C | 0.340323 | 1.54559 | 2.27058 |
| ANK1 | 1.15603 | 1.30185 | 2.93992 |
| SLC1A4 | 0.120133 | 2.07221 | 1.47072 |
| RUNDC3A | 0.959277 | 2.35837 | 6.91942 |
| UBE2O | 0.680411 | 1.23823 | 2.00606 |
| EPHB2 | 2.48838 | 2.21387 | 2.35018 |
| CHAC1 | 1.61862 | 5.8387 | 5.59268 |
| CUL3 | -0.386778 | -2.0602 | -2.56431 |
| EGR1 | 0.172197 | -1.48006 | -4.01243 |
| OLFM4 | -0.544573 | 3.70915 | 5.03879 |
| THRB | -0.605688 | -2.44198 | -1.28116 |
| PID1 | -1.34979 | -3.05623 | -3.29544 |
| ERG | -0.696365 | 1.07551 | 2.37743 |
| DCC | -0.552635 | 2.15265 | 1.37755 |
| WFS1 | 0.105034 | 2.53717 | 1.97808 |
| GPT2 | 0.865796 | 2.1548 | 1.86124 |
| ACHE | 4.29801 | 2.41374 | 4.21411 |
| SCML1 | -0.372215 | -2.04154 | -1.74665 |
| APCDD1 | -2.06936 | -2.81964 | -1.87089 |
| RAPGEF5 | -1.3762 | 3.14629 | 2.08954 |
| HBEGF | 0.415805 | -2.01738 | -1.52153 |
| JUP | 2.82262 | 1.69821 | 1.76041 |
| ST6GALNAC3 | -1.20764 | -3.17196 | -3.10205 |
| KLHL14 | -0.883998 | 2.41182 | 1.49526 |
| KCNN3 | -0.537071 | 2.60996 | 2.09689 |
| DPYD | -1.73108 | -3.11227 | -3.47812 |
| MPO | 0.539011 | 2.7178 | 3.06092 |
| IRF4 | 0.494462 | 2.4481 | 1.8745 |
| ALPL | 1.21616 | -2.77567 | -2.39883 |
| CGN | 1.22674 | 3.0005 | 2.32009 |
| FHDC1 | 2.16352 | 2.0789 | 5.0581 |
| YPEL3 | 0.993395 | 1.06343 | 2.08915 |
| PTGS2 | -0.165607 | -1.92686 | -2.77061 |
| CCL8 | 4.44052 | 3.6523 | 3.61067 |
| CREB5 | -0.769957 | -2.73672 | -2.88233 |
| NAMPT | -0.365603 | -2.18992 | -3.09998 |
| TCF3 | 0.519974 | 2.468 | 2.23456 |
| TMCC2 | 2.53577 | 1.47828 | 4.48364 |
| FAM107B | -0.351921 | -1.54617 | -2.3602 |
| SDC1 | 0.312248 | 4.61779 | 4.12224 |
| USP18 | 3.40844 | 2.3424 | 2.33648 |
| MRC2 | 0.931412 | NA | 2.21723 |
| MCAM | 1.93181 | 1.89238 | 2.30214 |
| XBP1 | 0.192979 | 2.1377 | 1.67379 |
| PMEPA1 | 0.496018 | 2.45901 | 1.89227 |
| DERL3 | -0.311855 | 3.15765 | 2.58458 |
| SLC2A1 | 1.03496 | 1.4201 | 2.62116 |
| ANKRD45 | 2.54135 | 2.36994 | 3.23534 |
| KANK2 | 2.01302 | 2.3826 | 3.76863 |
| ASPH | -0.489202 | -2.01803 | -1.90489 |
| TRAM2 | 0.458732 | 2.01794 | 1.85039 |

*^a^* LFC= Log2 Fold change
