# Supplementary material for: High-Throughput RNA Sequencing Analysis of Plasma Samples Reveals Circulating microRNA Signatures with Biomarker Potential in Dengue Disease Progression
Source: mSystems. 2020 Sep 15;5(5):e00724-20. doi: 10.1128/mSystems.00724-20 (PMC7498686; doi:10.1128/mSystems.00724-20)
Supplement: TABLE S4 [file mSystems.00724-20-st004.docx]

Table S4. Inverse expression of circulating microRNAs and their target genes in PBMCs

| Genes | *^a^* LFC_  DI | LFC_DWS | LFC_DS | miRNAs | LFC _DS  miRNA |
| --- | --- | --- | --- | --- | --- |
| SLC16A14 | -1.29 | 3.19 | 2.53 | hsa-miR-30d-5p  hsa-miR-30e-5p  hsa-miR-146a-5p  hsa-let-7i-5p  hsa-let-7f-5p  hsa-miR-199a-5p  hsa-miR-140-5p  hsa-miR-223-3p  hsa-miR-182-5p  hsa-miR-93-5p | -0.97149  -0.97082  -1.40178  -1.33618  -1.61255  -2.12123  -0.70867  -1.06123  -0.71089  -1.046 |
| EPHB2 | 2.48 | 2.21 | 2.35 | hsa-miR-30d-5p  hsa-miR-28-5p  hsa-miR-30e-5p  hsa-let-7f-5p  hsa-miR-199a-5p  hsa-miR-199b-5p  hsa-miR-140-5p  hsa-miR-93-5p | -0.97149  -1.31001  -0.97082  -1.61255  -2.12123  -2.09473  -0.70867  -1.046 |
| MPO | 0.539 | 2.717 | 3.060 | hsa-miR-181a-5p  hsa-miR-146a-5p  hsa-miR-99a-5p  hsa-miR-100-5p | -0.76278  -1.40178  -1.07404  -0.85381 |
| CUL3 | -0.386 | -2.060 | -2.56 | hsa-miR-423-5p  hsa-miR-103a-3p  hsa-miR-107  hsa-miR-501-5p  hsa-miR-877-5p  hsa-miR-486-5p  hsa-miR-92a-5p  hsa-miR-92b-3p | 1.501059  0.802567  0.993311  1.258664  1.08381  2.2528474  1.7444204  1.3324917 |
| E2F2 | 1.086 | 2.308 | 2.798 | hsa-miR-146a-5p  hsa-let-7i-5p  hsa-let-7f-5p  hsa-miR-26a-5p  hsa-miR-199a-5p  hsa-miR-199b-5p  hsa-miR-99a-5p  hsa-miR-100-5p  hsa-miR-182-5p  hsa-miR-127-5p  hsa-miR-26b-5p  hsa-miR-93-5p | -1.40178  -1.33618  -1.61255  -3.11589  -2.12123  -2.09473  -1.07404  -0.85381  -0.71089  -2.60382  -1.66266  -1.046 |
| OLFM4 | -0.544 | 3.709 | 5.038 | hsa-miR-28-5p  hsa-miR-146a-5p  hsa-let-7i-5p  hsa-let-7f-5p  hsa-miR-223-3p | -1.31001  -1.40178  -1.33618  -1.61255  -1.06123 |
| USP18 | 3.408 | 2.342 | 2.336 | hsa-miR-423-5p  hsa-miR-103a-3p  hsa-miR-107  hsa-miR-191-5p  hsa-miR-378a-5p  hsa-miR-501-5p  hsa-miR-484  hsa-miR-125b-5p  hsa-miR-93-5p  hsa-miR-340-5p  hsa-miR-16-5p | 1.501059  0.802567  0.993311  -2.58807  0.790618  1.258664  1.065252  1.218134  -1.046  -1.78712  0.4646 |
| NAMPT | -0.365 | -2.189 | -3.1 | hsa-miR-423-5p  hsa-miR-320a  hsa-miR-320b  hsa-miR-320c | 1.501059  1.678577  1.466815  1.741937 |
| CHAC1 | 1.618 | 5.838 | 5.592 | hsa-miR-26a-5p  hsa-miR-26b-5p | -3.11589  -1.66266 |
| CACNA1E | 0.366 | -2.645 | -2.60 | hsa-miR-486-5p  hsa-miR-423-5p  hsa-miR-103a-3p  hsa-miR-107  hsa-miR-320a  hsa-miR-320b  hsa-miR-320c  hsa-miR-877-5p  hsa-miR-122-5p | 2.252749  1.501059  0.802567  0.993311  1.678577  1.466815  1.741937  1.08381  4.310258 |
| MYBL2 | -0.226 | 2.528 | 2.276 | hsa-miR-30d-5p  hsa-miR-30e-5p  hsa-miR-182-5p  hsa-miR-127-5p | -0.97149  -0.97082  -2.60382  -0.71089 |
| MXD1 | 0.124 | -2.295 | -2.70 | hsa-miR-320a  hsa-miR-320b  hsa-miR-150-5p  hsa-miR-501-5p  hsa-miR-484  hsa-miR-345-5p  hsa-miR-320c  hsa-miR-877-5p  hsa-miR-375  hsa-miR-122-5p | 1.678577  1.466815  0.693807  1.258664  1.065252  0.816049  1.741937  1.08381  0.988967  4.310258 |

*^a^* LFC=Log fold change
